# Latitudinal trend in the reproductive mode of the pea aphid *Acyrthosiphon pisum* invading a wide climatic range

**DOI:** 10.1002/ece3.6536

**Published:** 2020-07-18

**Authors:** Sebastián I. Martel, Carmen G. Ossa, Jean‐Christophe Simon, Christian C. Figueroa, Francisco Bozinovic

**Affiliations:** ^1^ Departamento de Ecología Facultad de Ciencias Biológicas Center of Applied Ecology and Sustainability (CAPES) Pontificia Universidad Católica de Chile Santiago de Chile Chile; ^2^ Instituto de Biología Facultad de Ciencias Universidad de Valparaíso Valparaíso Chile; ^3^ UMR IGEPP INRAE Agrocampus Ouest Université de Rennes 1 Le Rheu France; ^4^ Instituto de Ciencias Biológicas Center for Molecular and Functional Ecology in Agroecosystems (CEMF) Universidad de Talca Talca Chile

**Keywords:** asexuality, invasion biology, maintenance of sex, pea aphid

## Abstract

The maintenance of sexuality is a puzzling phenomenon in evolutionary biology. Many universal hypotheses have been proposed to explain the prevalence of sex despite its costs, but it has been hypothesized that sex could be also retained by lineage‐specific mechanisms that would confer some short‐term advantage. Aphids are good models to study the maintenance of sex because they exhibit coexistence of both sexual and asexual populations within the same species and because they invade a large variety of ecosystems. Sex in aphids is thought to be maintained because only sexually produced eggs can persist in cold climates, but whether sex is obligate or facultative depending on climatic conditions remains to be elucidated. In this study, we have inferred the reproductive mode of introduced populations of the pea aphid *Acyrthosiphon pisum* in Chile along a climatic gradient using phenotypic assays and genetic‐based criteria to test the ecological short‐term advantage of sex in cold environments. Our results showed a latitudinal trend in the reproductive mode of Chilean pea aphid population from obligate parthenogenesis in the north to an intermediate life cycle producing both parthenogenetic and sexual progeny in the southernmost locality, where harsh winters are usual. These findings are congruent with the hypothesis of the ecological short‐term advantage of sex in aphids.

## Graphical abstract

Invasive population of *Acyrthosiphon pisum* in Chile is structured along a latitudinal transect. The southernmost one, where winters are harsh, is the only one that exhibits genetic and phenotypic features of sexual reproduction.

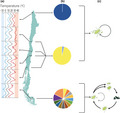

## 1 INTRODUCTION

Why and how sexual reproduction is maintained and widely spread is an intriguing question in evolutionary biology (Neiman, Lively, & Meirmans, 2017). Sex has evident short‐term energetic costs that go from the quantity of resources invested to produce males to the cost of mating activities (see Lehtonen, Jennions, & Kokko, 2012). The most evident consequence of these costs is demographic (Gibson, Delph, & Lively, 2017; Maynard‐Smith, 1978). However, although expensive, sexual reproduction is ubiquitous in nature. In fact, about 99.9% of the animal species reproduce sexually at least one time in their life cycle (Otto, 2008). Thus, the prevalence of sexual reproduction despite its costs is paradoxical (Bell, 1982; Maynard‐Smith, 1971, 1978).

Common attempts to explain the paradox of sex have focused on possible universal benefits that would compensate for these overwhelming costs. Up to now, more than 20 hypotheses have been proposed to explain the prevalence of sexual reproduction in natural populations (Butlin, 2002; Hartfield & Keightley, 2012; Kondrashov, 1993). Most of them can be grouped into two main categories: (a) Sex increases the rate of adaptive evolution generating new variants by gene recombination, or (b) Sex prevents the accumulation of deleterious mutations (Butlin, 2002). Nevertheless, the loss of sexual reproduction has been described in almost every eukaryotic lineage (Stelzer, 2015). Although it is believed that asexual lineages would be evolutionary dead ends, there are examples that demonstrate that sex is not strictly necessary and that these lineages can evolve without genetic reshuffle (Gorelick & Carpinone, 2009; Sentis et al., 2018). On the other hand, it has been proposed that sex can be retained by lineage‐specific mechanisms such as beneficial traits that have evolved within species and became associated with sexual reproduction (Gouyon, 1999). In this way, a universal explanation for the maintenance of sex would not be required (Gouyon, 1999; Stelzer, 2015).

**FIGURE 1:**
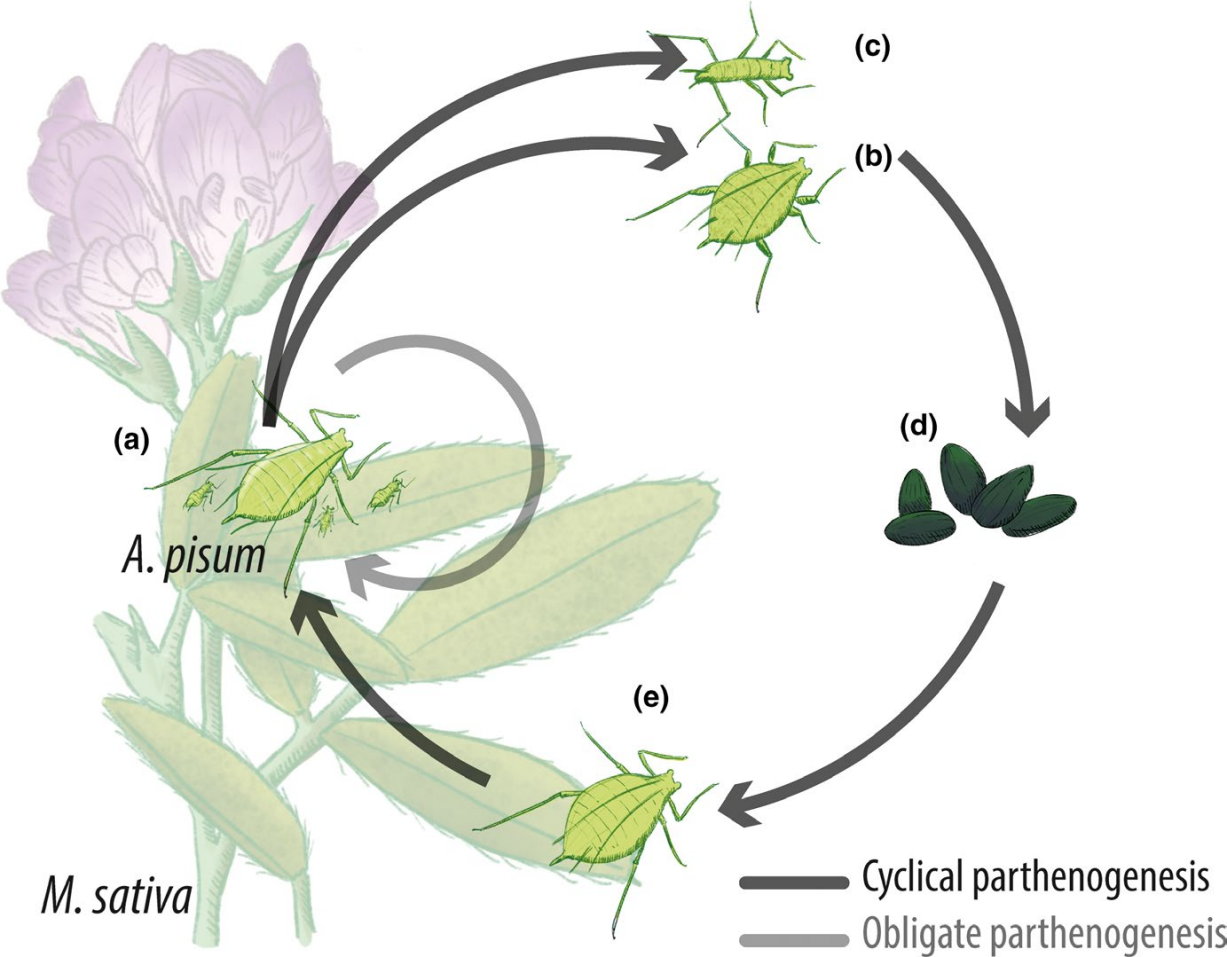
Illustration of the life cycle of *Acyrthosiphon pisum* aphids living on alfalfa (*Medicago sativa*), a ubiquitous perennial crop. The gray cycle represents obligate parthenogenesis (OP). The black cycle represents the ancestral life cycle of aphids, cyclical parthenogenesis (CP). In CP: (a) Asexual parthenogenetic females in spring and summer, (b) and (c) sexual females and males, respectively, at the beginning of fall. Also, a proportion of parthenogenetic females can be produced here. (d) Diapausing cold‐resistant eggs in winter. (e) Parthenogenetic fundatrix female at the beginning of the spring. Individuals in (a) and (c) can be winged but were excluded from the figure for simplicity

Aphids (Hemiptera: Aphididae) are a good system to investigate the evolution of sex, because (a) they show intraspecific coexistence of both cyclical parthenogenetic (CP lineages having obligate sex once in a year) and obligate parthenogenetic (OP lineages reproducing through permanent asexuality) populations (Figure 1), (b) they exhibit numerous transitions between different reproductive modes, and (c) these transitions are free of ploidy shifts (i.e., changes in the number of haploid chromosomes), which is not the case in most other asexual taxa (Simon, Rispe, & Sunnucks, 2002). In aphids, sex is thought to be maintained by environmental factors as only sexually reproducing aphids can produce diapausing eggs, the sole cold‐resistant form.

Aphids as a group comprise more than 5,000 species (van Emden & Harrington, 2007), some of them being highly invasive and recognized worldwide agriculture pests. There is a general thought that the success of most aphid species in novel environments relies on their capacity to shift permanently from CP to OP which confers the demographic benefits described above. In fact, in introduced ranges, only ~3% of the species retain CP (Figueroa, Fuentes‐Contreras, Molina‐Montenegro, & Ramirez, 2018). As a consequence, it is common to see that in invasive ranges there is a remarkable prevalence of only one or few asexual genotypes in large areas and during several years, the so‐called superclones (Vorburger, Lancaster, & Sunnucks, 2003). Nevertheless, few invasive aphid species maintain CP in the novel range, which depends on particular situations like (a) the availability of the host plant where sexual reproduction occurs, or (b) the magnitude of migrations between the source population(s) and the introduced range (Figueroa et al., 2018). Despite these cases, aphids have the capacity to frequently lose the sexual phase (Hardy, Peterson, & von Dohlen, 2015), making the question about the maintenance of sex even more paradoxical. In this regard, it has been hypothesized that the main reason for the maintenance of sex in aphids lies in lineage‐specific mechanisms linking sexual reproduction and cold‐resistant form production (Simon et al., 2002).

Chile is an ideal natural laboratory to study the maintenance of sexual reproduction in aphids. The Pacific Ocean and the Andes act as geographic barriers to natural events of introduction, making Chile a very isolated country. However, accidental introductions related to human activities may occur allowing to track invasions of sexual and asexual taxa and their respective evolutionary trajectories. In addition, Chile displays a great latitudinal span, comprising more than 40 latitudinal degrees (~4,500 km) from north to south, and various climatic conditions ranging from coastal deserts with constantly warm temperatures in the north to cold steppes with harsh winters in the south. Several studies about the clonal diversity and population structuring of introduced pest aphid populations have been conducted in Chile, including the grain aphid, *Sitobion avenae* (Figueroa et al., 2005), the peach‐potato aphid, *Myzus persicae* (Rubiano‐Rodríguez et al., 2014), the bird cherry‐oat aphid *Rhopalosiphum padi* (Rubio‐Meléndez, Barrios‐San Martín, Piña‐Castro, Figueroa, & Ramírez, 2019), and the pea aphid, *Acyrthosiphon pisum* (Peccoud et al., 2008), which represent some of the most invasive known aphid species. All these studies showed that asexual reproduction largely predominates in introduced aphid populations, as highlighted by low genotypic diversity and the occurrence of a few extremely frequent asexual clones that persist over years. However, these studies analyzed aphid populations only in climatic zones where parthenogenetic overwintering is possible (i.e., areas with mild winters).

Here, we used individuals of *A. pisum* sampled from six localities in Chile along a nearly 4,000 km transect and covering distinct climates, to test the short‐term ecological advantage of sex as a type of independent‐lineage mechanism for the maintenance of sex. We hypothesized that in the introduced range (Chile), the predominant reproductive mode would be OP in warm and temperate areas, but if aphids are present in colder areas, we predict that *A. pisum* would exhibit sexual reproduction which should allow populations to cope with freezing temperatures.

## 2 MATERIALS AND METHODS

### 2.1 Aphids sampling and laboratory rearing

Individuals of *A. pisum* were collected in spring and summer during 2016–2018 on the same host plant (i.e., alfalfa crops, *Medicago sativa*, L.) from three areas that feature different climates in Chile, hereafter named as North (apN), Central (apC), and South (apS) (Table 1 and Figure 3a). The sampling covered a latitudinal gradient of nearly 4,000 km (from 18° to 52° Lat. S), totalizing 15 alfalfa fields from six sampling localities (Table 1). Sampling was conducted on one to three alfalfa fields per latitude, each separated by at least three kilometers. Because *A. pisum* was reproducing asexually during the sampling period (whatever their reproductive mode, i.e., CP or OP) and to maximize the chance to sample aphids with different genotypes, the sampling within each alfalfa field was made taking only one individual per plant, surveying at least 6 plants per field separated by 3 m, thus totalizing 181 individuals across Chile (Table 1) with a comparable sampling effort in each field. Aphids were stored in 95% alcohol until DNA extraction. In order to perform phenotypic assessments of the reproductive mode of *A. pisum*, two individuals from each sampled latitude were taken alive to the laboratory to initiate stock parthenogenetic lineages (laboratory lines). Maintenance of separated laboratory parthenogenetic lineages was carried out on broad bean plants (*Vicia faba* L.) inside of 4 L transparent plastic buckets. Buckets were maintained in a climatic chamber at 20°C (±0.5°C) and long photoperiod (LD = 16:8) to ensure sustained parthenogenetic reproduction.

**TABLE 1:** Number of aphids sampled at each locality and associated geographic and climatic features

Locality	Climatic Zone	Latitude (°S)	Annual Temperature^a^ (°C)			Number of fields^b^	N Total^c^
			Mean	Max.	Min.		
Arica	apN	18	17.2	26.7	10	3	20
Valle del Elqui	apC	30	15.9	33	3.7	2	14
San Francisco	apC	33	14.6	30.3	2.4	1	12
Linares	apC	35	12.8	27	1.1	3	95
La Unión	apC	40	11.4	23	1.1	3	22
Punta Arenas	apS	52	6.8	17.7	−3.2	3	18
Total						15	181

^a^ Two years average annual temperature conditions (mean, maximum, and minimum) from spring 2016 to spring 2018 (from http://agromet.inia.cl).

^b^ Number of sampled alfalfa fields.

^c^ Total number of individuals obtained in each sampled latitude.

### 2.2 DNA extraction and MLG characterization

Highly polymorphic genetic markers (microsatellites) were used to identify multilocus genotypes (MLGs). We genotyped 181 field‐collected individuals at nine hypervariable microsatellite loci, namely AlA09M, ALB04M, AlB07M, AlB08M, AlB12M, ApF08M, ApH08M, ApH10M (Caillaud et al., 2004) and Ap03 (Kurokawa, Yao, Akimoto, & Hasegawa, 2004). All these microsatellites are among the most variable available for the pea aphid (Peccoud et al., 2008). Aphid total genomic DNA was extracted following the salting‐out protocol using proteinase‐K digestion and precipitation by ethanol (Sunnucks & Hales, 1996). Resulting DNA was resuspended in ultrapure water and set to a concentration of 30 ng/μl. Each locus was amplified separately with fluorescent‐labeled primers (6‐FAM or VIC) for each individual in 10 μl polymerase chain reactions (PCR). For every sample, the PCR mix included ultrapure water, approximately 60 ng of aphid DNA, PCR buffer at 1× final concentration, 2 mM of MgCl_2_, 0.5 U of Platinum Taq DNA polymerase (Invitrogen™), 200 mM of dNTP's, 125 pM of forward primer and 500 pM of reverse primer for each primer loci, and 500 pM of marked‐M13 primer (6‐FAM or VIC). The amplification steps were the following: (a) initial denaturation step at 94°C for 2 min; (b) 32 cycles consisting of a denaturation step at 94°C for 30 s, annealing at 60°C for AlA12M and AlB07M primers, or 56°C for all the other primers for 30 s, and an elongation step at 72°C for 45 s; (c) 8 more cycles of denaturation at 94°C for 30 s, annealing at 53°C for 30 s for all the primers, and the elongation at 72°C for 30 s; (d) a final elongation step at 72°C for 2 min.

Electrophoresis of amplified fragments was carried out by Macrogen Inc. using capillary sequencer ABI 3730Xls (Applied Biosystems). MLG characterization was made by checking visually the allele sizes of each sample using the software Geneious R8.0.5 (http://www.geneious.com, Kearse et al., 2012).

### 2.3 Characterization of reproductive mode

We assessed the reproductive mode of each subpopulation following two strategies: (a) by inducing sexual reproduction in individuals from laboratory parthenogenetic lineages; and (b) by inference from population genetic data.

#### 2.3.1 Phenotypic characterization

In *A. pisum*, as for most aphid species, the shift from clonal to sexual reproduction is triggered by an autumn‐like photoperiodic regime (shortening of daylength) and is a transgenerational process. In order to characterize the reproductive phenotype, individuals of each laboratory lineage from different latitudes reared under long‐day conditions (LD = 16:8, 20°C) were transferred to a climatic chamber at 15°C with a short‐day regime (LD = 12:12, following Frantz, Plantegenest, & Simon, 2006). After transfer into short‐day conditions, the progeny was checked daily until the third generation, looking for sexual females, males, mating individuals, and diapausing eggs, all signs of possible sexual reproduction.

#### 2.3.2 Population genetics survey

We followed the procedure recommended by Halkett, Simon, and Balloux (2005) for a genetic‐based inference of the reproductive mode in all three Chilean sampled zones of *A. pisum*. We estimated several population parameters for each of the six latitudes where aphids were collected, accounting for all the individuals from each alfalfa field. The proportion of distinct genotypes (*G*:*N* index) was calculated, where G is the number of different MLGs per subpopulation, and N the number of sampled individuals in that subpopulation. The mean number of alleles (*N*_a_), expected heterozygosity (*H*_e_), observed heterozygosity (*H*_o_) and deviation from expected heterozygosity under Hardy–Weinberg expectations (*F*_IS_) were obtained using GenAlEx v.6.1 (Peakall & Smouse, 2006, 2012). Finally, the linkage disequilibrium between loci was tested using Genepop v.4.2 (Raymond & Rousset, 1995; Rousset, 2008) performing 1,000 permutations on pairs of loci and considering all the clonal copies per MLG.

### 2.4 Analysis of genetic relatedness between MLGs

To estimate the relationship between MLGs, a matrix of shared allele distances (D_AS_) was constructed (Jin & Chakraborty, 1994) using the software POPULATIONS 1.2.31 (http://bioinformatics.org/~tryphon/populations/). Distance trees were calculated with the obtained values and then plotted as a neighbor‐joining tree using FigTree v1.4.3 (http://tree.bio.ed.ac.uk/software/figtree/).

## 3 RESULTS

### 3.1 MLGs characterization

Sixteen different MLGs were found among the 181 individuals sampled in Chile (Table 2). Genotypic diversity was the highest in the southernmost locality (apS, 52°S, Table 3) where 14 out of 16 MLGs were found. In contrast, localities in central Chile (apC, 30°S–40°S) exhibited only two MLGs hereafter named APG2 and APG3 (see Table 2). One of them, the APG3 genotype, accounted for 96% of the total sampled individuals in central Chile, whereas APG2 was found in the remaining 4% in the same region. Interestingly, one individual from the apS sample had an APG3 genotype. Finally, in the northern locality (apN, 18°S), all the individuals belonged to APG1, being the only MLG found in that locality. Moreover, APG1 and APG2 MLGs are genetically similar, differing only by the additions of a pair of nucleotides in two alleles (see Table 2). Therefore, it can be assumed that both genotypes descent from a common ancestor, and in consequence, hereafter both are referred to as the APG1/2 complex.

**TABLE 2:** Allele combinations at nine microsatellite loci for 16 multilocus genotypes (MLGs) found in 181 pea aphid sample along Chile

MLG	Microsatellite locus								
	ApF 08	ALB 04	ALB 08	ALB 12	ALB 07	ApH 10	Ap 03	ApH 08	ALA 12
APG1	180/192	266/268	294/302	328/334	136/172	214/214	252/260	266/268	449/457
APG2	180/192	266/268	294/302	328/334	136/170	214/216	252/260	266/268	449/457
APG3	182/188	266/268	276/290	320/344	136/152	202/212	260/260	268/284	435/459
APG4	184/190	262/268	294/304	318/334	136/158	200/216	260/260	266/268	451/457
APG5	182/194	262/266	302/304	320/328	136/158	216/216	260/260	268/270	449/451
APG6	182/182	262/268	294/304	320/328	136/158	202/216	260/260	266/268	451/457
APG7	182/194	262/268	294/304	318/328	158/172	214/216	260/260	268/268	451/457
APG8	190/194	266/268	294/302	320/328	136/136	202/216	260/260	268/270	449/457
APG9	190/194	266/268	302/304	328/328	136/142	202/216	260/260	268/268	435/449
APG18	182/194	266/268	302/304	320/334	136/160	198/202	260/260	266/284	451/461
APG11	182/194	266/266	302/302	320/334	136/170	212/216	260/260	266/270	449/467
APG12	182/194	266/268	302/304	318/328	142/158	216/216	260/260	268/268	449/451
APG13	182/192	262/268	304/304	318/334	142/158	214/216	260/260	268/268	435/451
APG14	182/194	262/266	302/304	318/334	142/158	216/216	260/260	266/268	435/449
APG15	190/194	266/268	302/304	328/328	136/142	202/216	260/260	268/268	435/449
APG16	182/194	268/268	302/302	318/328	136/160	198/216	260/260	268/284	449/461

Note

Numbers represent allele sizes (bp).

**TABLE 3:** Genetic diversity estimators by sampling location

Locality	*N*_a_	*H*_o_	*H*_e_	*F*_IS_	LD^b^	*G*:*N*
Arica	1.89	0.89	0.44	−1^a^	—	0.05
Valle del Elqui	1.89	0.89	0.44	−1^a^	—	0.07
San Francisco	1.89	0.89	0.44	−1^a^	—	0.08
Linares	3.56	0.9	0.49	−0.75^a^	28/28	0.02
La Unión	1.89	0.89	0.44	−1^a^	—	0.05
Punta Arenas	5	0.73	0.6	−0.2^a^	26/28	0.78
All population	2.69	0.86	0.5	—		0.09

Note

Mean number of alleles (*N*_a_), observed heterozygosity (*H*_o_), expected heterozygosity (*H*_e_), deviation from expected heterozygosity under Hardy–Weinberg expectations (*F*_IS_), linkage disequilibrium (LD) and *G*:*N* Index for each sampling site.

^a^ Indicates departures from Hardy–Weinberg Equilibrium (*p* = .001).

^b^ No possible comparisons between loci were excluded because the clonal nature of the samples. Only possible comparisons (out of 36 in total) are shown.

### 3.2 Phenotypic characterization of reproductive mode

**FIGURE 2:**
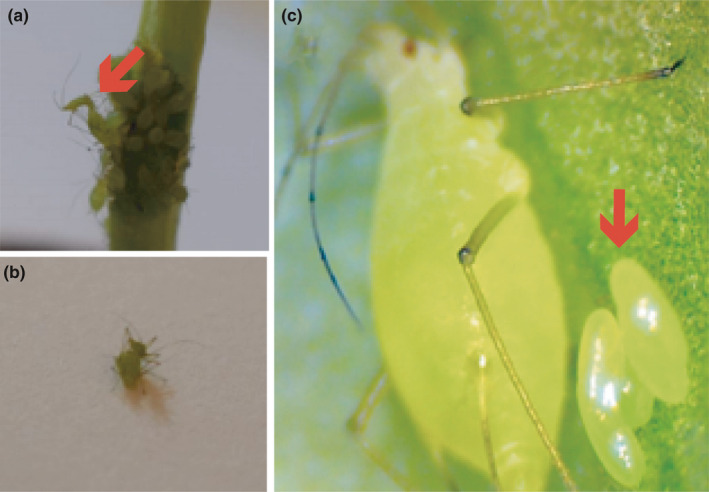
Pictures of pea aphid individuals collected in Punta Arenas and taken back alive to the laboratory. (a) and (b) mating events between sexual males and sexual females. (c) immature diapausing cold‐resistant eggs

The response to short‐day conditions that is known to trigger the production of sexual forms in aphids was assessed on 12 *A. pisum* lineages coming from six sampled localities and belonging to five MLGs, in order to characterize their reproductive mode in Chile. The APG1/2 complex, which dominates in northern Chile and represents a small fraction of individuals in Central Chile, was constantly found as strictly asexual (only parthenogenetic forms were produced in response to short‐day condition) in the three tested lineages. The genotype APG3, which was mainly restricted to central Chile (with the exception of a single individual found in the southern collection, apS), consistently produced males along with parthenogenetic females, but no sexual females in all the seven tested lineages (Table 4). In addition, no mating events or eggs were visually recorded for these lineages. By contrast, APG4 and APG13, two genetically distinct MLGs coming from the southernmost latitude, were both able to produce sexual females and males, and mating events and eggs were also recorded (Table 4 and Figure 2).

**TABLE 4:** Presence/absence of sexual phenotypes per locality after rearing different aphid genotypes under laboratory conditions that induce sexuality in the pea aphid

Locality	MLG	Males	Sexual Females	Mating Events	Eggs
Arica	APG1	✕	✕	✕	✕
	APG1	✕	✕	✕	✕
Valle del Elqui	APG3	✓	✕	✕	✕
	APG3	✓	✕	✕	✕
San Francisco	APG3	✓	✕	✕	✕
	APG3	✓	✕	✕	✕
Linares	APG2	✕	✕	✕	✕
	APG3	✓	✕	✕	✕
La Unión	APG3	✓	✕	✕	✕
	APG3	✓	✕	✕	✕
Punta Arenas	APG4	✓	✓	✓	✓
	APG13	✓	✓	✓	✓

Note

Observed (✓)/ not observed (✕) sexual phenotypes for two laboratory lineages coming from each sampled locality after being reared under short photoperiod conditions (LD = 12:12 and 15°C).

### 3.3 Genetic characterization of the reproductive mode

The clonal diversity computed as *G*:*N* index was extremely low in five out of the six sampled localities (ranging from 0.05 to 0.08), whereas in the southernmost locality the *G*:*N* index was 0.78. The genetic diversity for all localities is summarized in Table 3. Our results indicate that all six latitudinal subpopulations show deviations from Hardy‐Weinberg equilibrium (*p* = .001, Table 3), having a low number of alleles per locus (a total mean of 2.685). The observed heterozygosity was higher than expected (*F*_IS_ = 0.001) for all six subpopulations. Nevertheless, although statistically different, the closest *H*_o_ to *H*_e_ (0.73 and 0.60, respectively) was found in the southernmost locality (apS). Finally, evidence of linkage disequilibrium was found in 26/28 (out of 36) comparisons between loci in the southernmost region (*p* < .05, Table 3). Comparisons were not possible in some or all the loci combinations for all the six sample localities because of the clonal nature of the samples and because the alleles for some loci were the same for all individuals.

### 3.4 Genetic relatedness between MLGs

**FIGURE 3:**
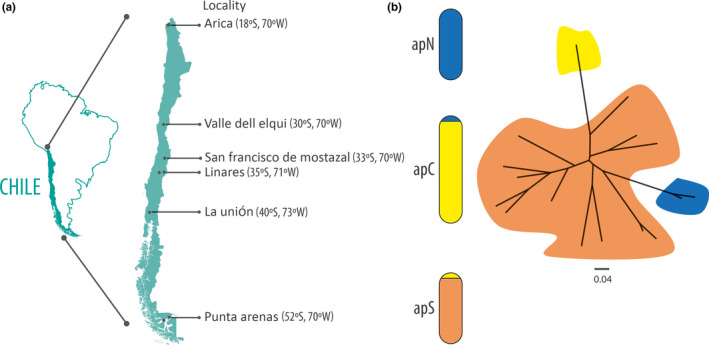
(a) Map of the Chilean sampled localities with their approximate coordinates in grades. (b) Unrooted neighbor‐joining tree based on the allele shared distance at nine microsatellites showing the genetic relatedness between the 16 Chilean *Acyrthosiphon pisum* MLGs. Different colors represent the MLGs found in each climatic zone

The output of the matrix of shared allele distances is shown in Figure 3 B as a neighbor‐joining tree. The branch's supports were not higher than 0.36 in any case. Nevertheless, the figure shows that MLGs found in apS are close to both the APG1/2 and APG3 genotypes.

## 4 DISCUSSION

**FIGURE 4:**
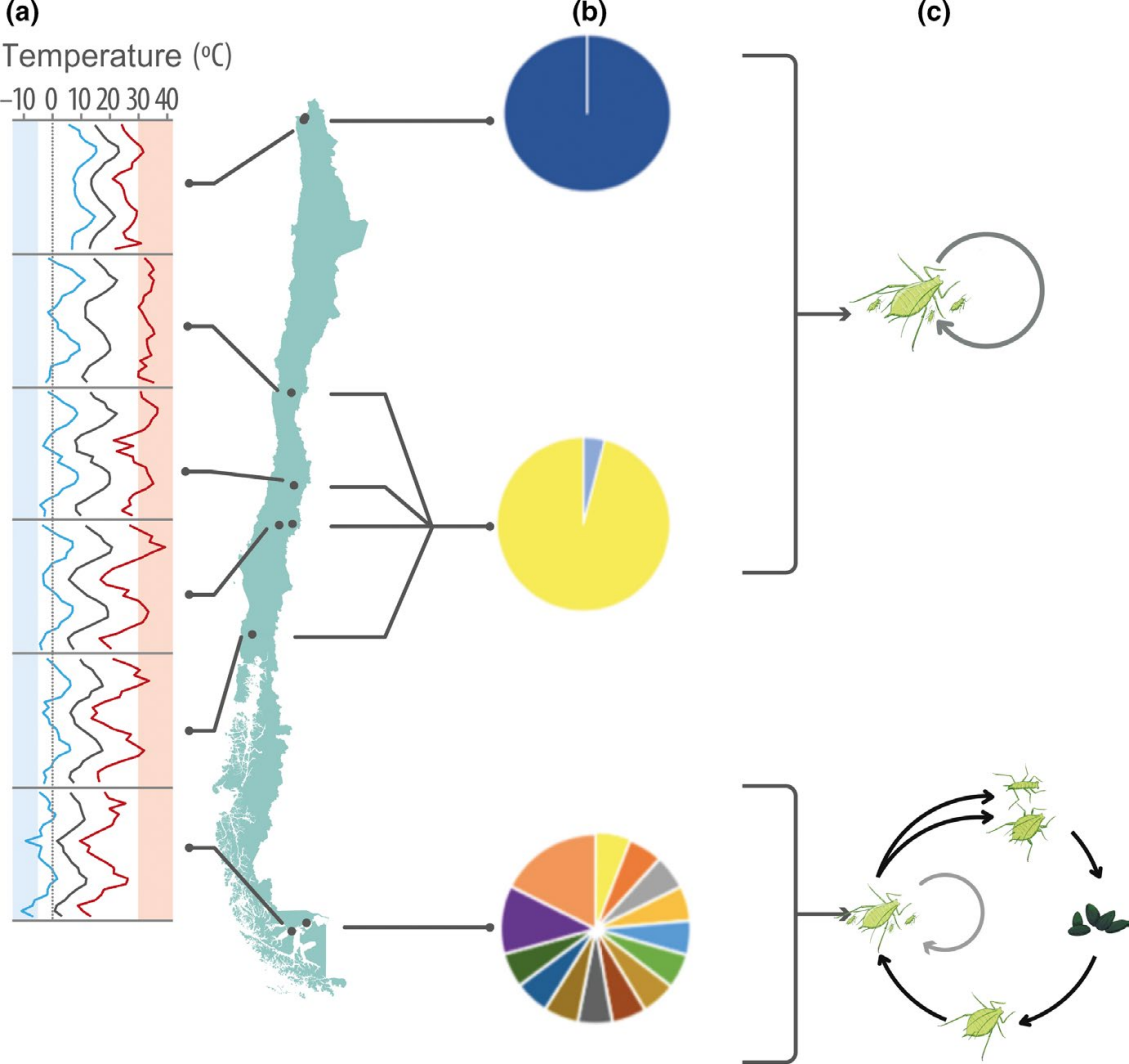
General summary outline. (a) Monthly Mean (black), Max. (red), and Min. (blue) temperatures (from http://agromet.inia.cl) for a 2 years period (from spring 2016 to spring 2018) for each sampled latitudinal locality (on the green map). Pale blue and pale red columns represent −5°C and 30°C, respectively. (b) Pie charts showing the proportion of the MLGs found in each climatic zone. Each color represents a unique MLG. In the south, there is a copy of APG3 (in yellow), the same MLG found in Central Chile. (c) Proposed reproductive mode for each subpopulation considering both phenotypic and genetic assays. Sufficient evidence for cyclical parthenogenesis was found only in the southern locality

In this study we used *A. pisum* aphids collected from alfalfa fields located in a latitudinal transect of about 4,000 km and covering various climates, to test for an ecological short‐term advantage of sex in cold environmental conditions. Both phenotypic and genetic data provided evidence for signs of sexual reproduction only in the southernmost climatic zone, where winter conditions are harsh and extreme in comparison to northern localities (Table 1 and Figure 4a). The highest clonal diversity, the closest observed to expected heterozygosity, and the production of cold‐resistant eggs only by lineages from the southernmost locality provides evidence for sexual reproduction in an invasive range and its link with cold conditions. In contrast, obligate parthenogenesis with no male production, and obligate parthenogenesis but with male production were found in northern and central areas of Chile, respectively (Tables 3 and 4; Figures 2 and 4).

The main advantage of OP lineages over CP ones is demographic: In mild winters areas where they can survive, OP lineages can continuously produce a large offspring number due to parthenogenesis and telescoping of generations (Simon et al., 2002; Simon, Stoeckel, & Tagu, 2010). In contrast, in addition to paying the costs associated with sexual reproduction, CP lineages overwinter as diapausing eggs, which require both a long developmental time (around 100 days) and a low‐temperature period (between 0 and 4°C) to ensure their correct development and hatching (Shingleton, Sisk, & Stern, 2003). These temperature requirements are not met in many mild‐winter areas, thus giving a competitive advantage to the OP lineages over the CP ones. This advantage becomes even more evident in introduced areas, with generally benign climates, in which only 3% retains CP (Figueroa et al., 2018).

Geographic patterns of sexuality/asexuality have been reported before (e.g., Burke & Bonduriansky, 2018; Frantz et al., 2006; Simon et al., 1999; Tilquin & Kokko, 2016). However, there are few studies using such an extensive transect across geographically isolated areas as presented here (but see Kanbe & Akimoto, 2009; Mackay, Lamb, & Smith, 1993). Natural barriers allow evaluating isolated populations in their non‐native range because genetic exchange with other populations seems unlikely. These kinds of systems are ideal because they act as natural laboratories to study the evolution of sexuality and its maintenance as well as the evolutionary and ecological forces shaping population genetic structure.

The pattern of reproductive mode variation and genetic structure found here could be explained by three nonmutually exclusive hypotheses: (a) independent introductions of genotypes with distinct reproductive phenotypes, (b) a reproductive mode transition from CP to OP in previously introduced populations and, (c) a reverse transition from OP to CP. The first scenario is supported by the work of Peccoud et al. (2008), which reported multiple events of introductions for the pea aphid in Chile, presumably from Western Europe. In this context, the introduction of invasive species of agricultural importance is more likely to occur from the central region of Chile (Estay, 2016). This is because most agricultural activities are carried out in the Central region of Chile due to its privileged climatic conditions, as well as major ports, airports and ground crossings for international trade are located in this area. On the other hand, the north (arid deserts) and south (cold steppes) regions are less suitable for agriculture. Yet, multiple introductions could hardly explain the diversity of genotypes in the south (apS), because of the scarcity of both agricultural and commercial activities in this region. Here, the high genetic diversity revealed in our work is better explained by sexual reproduction of local populations derived from one or a few introduced CP lineages. The second scenario (i.e., transition from CP to OP) postulates the introduction of one or a few CP lineages that were heterozygous for the locus controlling reproductive mode variation (Jaquiéry et al., 2014). Under the environmental conditions in southern Chile, these CP lineages may produce sexual forms and recombined progenies, some of them inheriting OP alleles. Then, the so‐produced OP lineages may spread to warmer areas where their rapid multiplication by parthenogenesis is favored. Lastly, a transition from OP to CP, although unlikely, cannot be discarded. Under this scenario, a complex trait like the capacity to produce sexual forms would have been gained by a reverse mutation in an OP lineage, thus restoring the CP phenotype. Then, this revertant would have fit with the environmental conditions that trigger and favor the CP phenotype (i.e., southern Chile). In the current state of our knowledge and observations, we cannot favor one or the other of these three hypotheses. In addition, the patterns of clonal and genetic diversities observed here may result from a combination of hypothetical events. For instance, the APG3 genotype, which shows the larger distribution across Chile and which is able to produce males under laboratory conditions, may have mated with CP females from southern populations (apS), thus resulting in an admixed progeny of CP and OP genotypes that maintain reproductive mode variation at a country scale. However, further studies are necessary to elucidate possible routes of introduction and spread of *A. pisum* genotypes in Chile.

The extremely low genetic diversity in the northern and central regions may also result from additional factors than few introduction events of asexually reproducing lineages. Indeed, selective agents as pesticides, extreme thermal events, or biotic interactions among others, can contribute to the paucity in clonal diversity (Frantz et al., 2006; Gilabert et al., 2009). As a consequence, very rapid changes in the genetic structure of populations could arise from an overrepresentation of the most fitted clonal lineages (as few as one or two: e.g., APG1/2 and APG3), which are also referred to as superclones (Figueroa et al., 2018; Vorburger et al., 2003). As an example, in the study of Brévault, Carletto, Tribot, Vanlerberghe‐Masutti (2011) on the cotton aphid *A. gossypii*, they found that in the cotton‐producing regions of west and central Africa only one of the two overrepresented genotypes was prevalent (>90%) in spite of the equal or even high performance of the second clone (accounting for <10%) on plants not sprayed with insecticides.

Causes for the loss of sexuality in aphids are not well understood. It has been hypothesized that aphid species have the capacity to lose the sexual phase by mechanisms that include contagious asexuality via pre‐existing parthenogenetic lineages or spontaneous mutations in the gene(s) controlling the production or the function of sexual forms (see Jaquiéry et al., 2014; Simon et al., 2002). Indeed, some species have lost sex completely (Hardy et al., 2015). Here, in APG1/2 we did not find any sign of sexual reproduction. The first alfalfa fields in Chile were established long before the 19th century (Gay, 1865) but the first record of *A. pisum* dates from the early 70s (Zúñiga, Franca, Norambuena, & Quiroz, 1985). The maximum number of generations of *A. pisum* in all these years (~50 years) at an optimal temperature and with parthenogenetic reproduction system could be approximately 2,500 (following Siddiqui, Barlow, & Randolph, 1973). Hence, it is likely that *A. pisum* populations living in habitat with benign climates could have lost sexuality in 2,500 generations or even fewer.

Along with mutations, sex is usually recognized as the main source of new additive genetic variance that drives eukaryotic evolution. Conversely, sex can act as a constraint on genomic and epigenetic variation, thereby limiting adaptive evolution (Gorelick & Heng, 2011; Verhoeven & Preite, 2014; Wilson, Sunnucks, & Hales, 2003). In this context, in lineages that have gained the ability to break away from sex (as aphids), the prevalence of sexual reproduction events can be due to some lineage‐specific factors. According to our results, sexuality in *A. pisum* is mainly found in higher latitudes where harsh winters are usual (mean winter minimum T° = −5.2°C for apS), not being present in warm and mild climates (mean winter minimum T° = 8.9 and −0.2°C for apN and apC respectively). In this regard, sexuality for this species could be maintained only as a strategy to thrive in areas with unfavorable environmental conditions.

Summarizing, the phenotypic assays and the genetic data analyses show that asexual reproduction dominates *A. pisum* populations from northern and central Chile where winters are mild, while signs of sex and recombination were only observed in populations from the south where winters are harsh. These results are congruent with the hypothesis of an ecological short‐term advantage of sex, a type of lineage‐specific mechanism for the maintenance of sexual reproduction. Although the temperature is an important driver of reproductive mode variation in aphids, other ecological variables could also have some effects on the maintenance of sexuality (e.g., environmental heterogeneity or biotic interactions), for which more studies are necessary to support the lineage‐specific hypothesis of maintenance of sex.

## CONFLICT OF INTEREST

The authors declare no competing interest.

## AUTHOR CONTRIBUTIONS

**Sebastián I. Martel:** Conceptualization (equal); formal analysis (lead); investigation (lead); methodology (lead); visualization (lead); writing – original draft (lead); writing – review and editing (equal). **Carmen G. Ossa:** Formal analysis (supporting); writing – original draft (supporting). **Jean‐Christophe Simon:** Conceptualization (equal); formal analysis (equal); resources (supporting); writing – review and editing (equal). **Christian C. Figueroa:** Conceptualization (equal); resources (supporting); writing – review and editing (equal). **Francisco Bozinovic:** Conceptualization (equal); funding acquisition (lead); resources (lead); supervision (lead); writing – review and editing (equal).

## Data Availability

The data that support the findings of this study (i.e., sampling locations and microsatellite genotypes) are available in a public data repository, Dryad https://doi.org/10.5061/dryad.3xsj3txbk.
